# Inhibition of serotonin-Htr2b signaling in skeletal muscle mitigates obesity-induced insulin resistance

**DOI:** 10.1038/s12276-025-01460-x

**Published:** 2025-06-02

**Authors:** Suhyeon Park, Hyeongseok Kim, Soyeon Shin, Yeongmin Kim, Yunwon Kang, Hye-Na Cha, So-Young Park, Sangkyu Park, Chang-Myung Oh

**Affiliations:** 1 Department of Biomedical Science and Engineering, Gwangju Institute of Science and Technology, Gwangju, Republic of Korea; 2 Department of Biochemistry, College of Medicine, Chungnam National University, Daejeon, Republic of Korea; 3 Department of Physiology, College of Medicine, Yeungnam University, Daegu, Republic of Korea; 4 Senotherapy-based Metabolic Disease Control Research Center, College of Medicine, Yeungnam University, Daegu, Republic of Korea; 5 Department of Biochemistry, Yonsei University Wonju College of Medicine, Wonju, Republic of Korea; 6 Organelle Medicine Research Center, Yonsei University Wonju College of Medicine, Wonju, Republic of Korea

**Keywords:** Metabolic syndrome, Transcriptomics

## Abstract

Obesity-induced insulin resistance is a major cause of metabolic disorders, including type 2 diabetes mellitus. Although peripheral serotonin (5-hydroxytryptamine, 5-HT) has been implicated in energy balance and metabolism, its effect on skeletal muscle insulin sensitivity remains unclear. Here we identified the 5-HT receptor 2b (Htr2b) as a critical regulator of insulin sensitivity and energy metabolism in the skeletal muscle. Using genetic and pharmacological approaches, we showed that muscle-specific *Tph1*-knockout (*Tph1* MKO) mice fed a high-fat diet exhibited reduced body weight, increased lean mass and improved glucose tolerance compared with wild-type mice. The pharmacological inhibition of Htr2b in myotubes reversed palmitate-induced insulin resistance and increased glycolytic activity. Moreover, muscle-specific *HTR2b*-knockout (*HTR2b* MKO) mice exhibited improved glucose uptake, insulin sensitivity and overall metabolic health under high-fat-diet-induced obesity. Mechanistically, both *Tph1* MKO and *Htr2b* MKO mice showed increased phosphorylation of AKT and AMPK, indicating improved insulin sensitivity and energy metabolism in the skeletal muscle. These findings demonstrate that 5-HT-Htr2b signaling negatively regulates insulin sensitivity and energy metabolism in skeletal muscles, providing new insights into the role of peripheral serotonin in muscle metabolism and potential therapeutic targets for metabolic disorders.

## Blocking serotonin receptor 2b enhances muscle insulin sensitivity

Obesity is a growing global health issue, leading to diseases such as diabetes and heart problems. Researchers are exploring new treatments targeting skeletal muscle, which plays a key role in managing blood sugar. They used mice with diet-induced obesity to study the effects of blocking serotonin signaling in skeletal muscle. They used genetic techniques to create mice lacking specific serotonin receptors in their muscles. They also used drugs to block these receptors in muscle cells grown in the lab. The study found that blocking serotonin improved insulin sensitivity and reduced fat buildup in muscles. This was linked to increased activity of proteins like AKT and AMPK, which help regulate energy use and storage in cells. They conclude that targeting serotonin signaling in muscles could be a promising approach to treat obesity-related metabolic issues. This summary was initially drafted using artificial intelligence, then revised and fact-checked by the author.

## Introduction

Obesity is now widely recognized as a global public health crisis, contributing significantly to the rise in metabolic disorders, including insulin resistance, type 2 diabetes mellitus and cardiovascular disease, as well as the incidence and mortality of many cancers^1^. The global prevalence of obesity nearly tripled between the years 1975 and 2016^2^. Moreover, from 1999 to 2018, the prevalence of obesity in the USA has increased from 30.5% to 42.4%^3^. In Korea, the overall prevalence of obesity was 38.4% in 2021, a 1.27-fold increase from 30.2% in the year 2012^4^.

Skeletal muscle plays a key role in obesity-related metabolic disorders, as it is the primary site for insulin-stimulated glucose uptake, accounting for 80% of postprandial glucose clearance from the bloodstream^5,6^. Therefore, impaired insulin signaling in skeletal muscle is a hallmark of obesity-induced insulin resistance and metabolic dysfunctions^6^. Therefore, skeletal muscle-targeted therapeutics have been proposed for the treatment of metabolic diseases such as obesity and diabetes^7,8^.

Serotonin (5-hydroxytryptamine, 5-HT) is a monoamine neurotransmitter^9^. Accumulating evidence suggests that 5-HT and 5-HT receptors (Htrs) also play important roles in the regulation of energy balance, glucose homeostasis and lipid metabolism in peripheral tissues^10,11^. Notably, 5-HT regulates pancreatic beta cell mass, insulin secretion^12^, energy metabolism in both white and brown adipose tissue^13,14^ and hepatic steatosis and regeneration in the liver^15^. While studies have extensively investigated peripheral 5-HT in metabolic dysfunction, the exact role of 5-HT and its receptor in mediating downstream signaling pathways in skeletal muscle remains underexplored. Elucidation of these mechanisms is critical for the development of effective therapeutic strategies to restore insulin sensitivity and mitigate metabolic complications.

In this study, we investigate the effects of inhibiting 5-HT signaling on skeletal muscle insulin sensitivity in a mouse model of diet-induced obesity. We hypothesize that 5-HT inhibition could restore normal insulin signaling and glucose uptake in skeletal muscles through the 5-HT receptor 2b (Htr2b), thereby alleviating obesity-induced insulin resistance. Using a combination of genetic and pharmacological approaches, we aim to elucidate the underlying mechanisms by which Htr2b inhibition modulates skeletal muscle metabolism and to explore its potential as a therapeutic target for alleviating metabolic diseases associated with obesity.

## Methods

### Reagents

SB204741, insulin (bovine pancreas), d-glucose, AICAR and Compound C were purchased from Sigma-Aldrich. The antibodies used in this study were as follows: rabbit AMPKα (Cell Signaling Technology, #5831), rabbit AMPK β1 (Cell Signaling Technology, #4178), mouse AMPK gamma 1 (Proteintech, 67182-1-Ig), rabbit phospho-AMPKα1 (Ser485)/AMPKα2 (Ser491) (Cell Signaling Technology, #4185), rabbit phospho-AMPKα (Thr172) (Cell Signaling Technology, #2531), rabbit acetyl-CoA carboxylase (Cell Signaling Technology, #3676), rabbit phospho-acetyl-CoA carboxylase (Ser79) (Cell Signaling Technology, #3661), rabbit AKT (Proteintech, 10176-2-AP), rabbit Phospho-AKT (ser473) (Cell Signaling Technology, #9018), rabbit CPT1B (Proteintech, 22170-1-AP), rabbit ATGL (Cell Signaling Technology, #2138), mouse GLUT4 (Cell Signaling Technology, #2213S), rabbit Phospho-HSL (Ser565) (Cell Signaling Technology, #4137), mouse GAPDH (Abbkine, ABL1020), rabbit HSL (Cell Signaling Technology, #4107), rabbit hexokinase II (Cell Signaling Technology, #2867), rabbit IRS-1 (Cell Signaling Technology, #2382), rabbit Phospho-IRS-1 (Ser1101) (Cell Signaling Technology, #2385), rabbit Phospho-LKB1 (Ser428) (Cell Signaling Technology, #3482), rabbit LKB1 (Cell Signaling Technology, #3047), rabbit SIRT1 (Abcam, ab189494), rabbit TPH1 (Invitrogen, MA5-32209), rabbit Phospho-PI3 Kinase p85 (Tyr458)/p55 (Tyr199) (Cell Signaling Technology, #4228), mouse PI3 Kinase p85 Alpha (Proteintech, 60225-1-Ig), mouse Total OXPHOS Rodent WB Antibody Cocktail (Abcam, ab110413) and rabbit -5-HT-2B (Abcam, ab221181).

### Cell cultures and treatments

Mouse C2C12 myoblasts were obtained from the Korean Cell Line Bank (Seoul, South Korea) and cultured in high-glucose Dulbecco’s modified Eagle’s medium (DMEM) (Gendepot, CM002-050) supplemented with 10% (v/v) fetal bovine serum and 1% (w/v) penicillin-streptomycin (HIMEDIA, A001-100). The cultures were maintained in a humidified incubator at 37 °C with 5% (v/v) CO_2_. To initiate differentiation, the cells were grown to 70–80% confluence and incubated in DMEM supplemented with 2% (v/v) horse serum (New Zealand origin, 16050122) for 4–6 days with daily medium changes. The fully differentiated myotubes were used in subsequent experiments. A 100 mM palmitate stock solution (Sigma P0500) was prepared in 0.1 M NaOH by heating at 70 °C in a shaking water bath. A 10% (w/v) fatty acid-free bovine serum albumin (BSA) solution (Sigma, #A7030) was prepared using double-distilled water (ddH_2_O) at 55 °C. A 5 mM palmitate/10% (w/v) BSA stock solution was then prepared by mixing 50 µl of the 100 mM palmitate solution with 950 µl of the 10% (w/v) BSA solution, while maintaining the temperature at 55 °C. The *Tph1*-knockout (KO) cell lines were generated using a CRISPR–Cas9 system, as previously described^16^.

For *Tph1* KO, two oligos (5′-CACCGTGGCGTGCGACTTCAGCAGG-3′ and 5′-AAACCCTGCTGAAGTCGCACGCCAC-3′) were inserted into lentiCRISPRv2 puro (Addgene, #98290). *HTR2b* expression was knocked down using siRNA (5′-ATCGAGACTGATGTGATTAAT-3′).

Glucose uptake by the differentiated C2C12 myotubes was measured using the 2-NBDG assay (Abnova, KA6077), employing a fluorescent d-glucose analog. The myotubes were preincubated for 8 h in DMEM containing 0.125 g l^−1^ glucose and 0.25% BSA. The cells were then incubated with 2-NBDG (33 μg ml^−1^) at 37 °C for 1 h. The uptake of 2-NBDG was stopped by removing the incubation medium and washing the cells with phosphate-buffered saline (PBS) to remove free 2-NBDG. Finally, the 2-NBDG fluorescence levels were determined using a fluorescence microscope (Nexcope) and quantified using ImageJ software.

### Animals and diets

To generate muscle-specific *Tph1*-KO (*Tph1* MKO) mice, *Tph1*^*flox/flox*^ mice (MGI:6271993) were crossed with *HSA*-Cre79 mice (MGI:2447635). To generate muscle-specific *Htr2b*-KO mice, *Htr2b*^*flox/flox*^ mice (MGI: 3837400) were crossed with *HSA*-Cre79 mice. The mice were housed in climate-controlled, specific pathogen-free barrier facilities under a 12-h light–dark cycle, with food and water provided ad libitum. All experimental protocols were approved by the Institutional Animal Care and Use Committee of the Gwangju Institute of Science and Technology (no. GIST-2020-105). The mice were fed either a standard chow diet (Research Diet D10001) or a high-fat diet (HFD) (Research Diet D12492, 60% fat calories). The metabolic rate was measured in individual chambers using an eight-chamber, open-circuit Oxymax/Columbus Instruments Comprehensive Lab Animal Monitoring System, as previously described^14^.

### GTT and ITT

The glucose tolerance test (GTT) and insulin tolerance test (ITT) were performed as previously described^14^. For the GTT, the mice were administered 2 g kg^−1^ d-glucose in PBS after overnight fasting. For the ITT, mice were injected intraperitoneally with insulin (1 U/kg) after a 6-h fast. The blood samples were collected from the tail vein at 0, 15, 30, 45, 60, 90 and 120 min after injection.

### Protein extraction and western blot analysis

The scraped cells were homogenized using Φ2.8 mm ceramic beads (Bioprep-A13). The tissue samples were homogenized using a mortar and pestle. Subsequently, 20 mg of the resulting homogenized powder was collected in a microcentrifuge tube for protein analysis. Both the cell and tissue samples were lysed using RIPA buffer (Intro IBS-BR002) supplemented with a phosphatase inhibitor cocktail (GDP, 93300-005), shaken for 1 h at 4 °C in a refrigerated shaker (MyLab, SLRM-3) and then centrifuged at 12,000 rpm for 10 min at 4 °C. The protein concentrations were quantified using the DC Protein Assay Kit I (Bio-Rad, 5000111). The samples containing 25–50 μg of protein were heated in Laemmli buffer (L1100-001) at 95 °C for 5 min. The proteins were separated via sodium dodecyl sulfate–polyacrylamide gel electrophoresis using a 10–12% gradient gel and transferred to polyvinylidene fluoride membranes (West-Q polyvinylidene fluoride membrane) using a wet electroblotting system. The membranes were blocked with 3% BSA for 1 h at room temperature and incubated with primary antibodies overnight. After washing with TBST, the membranes were incubated with horseradish peroxidase-conjugated secondary antibodies for 1 h at room temperature. The proteins of interest were detected using an enhanced chemiluminescence solution with the WSE-6200 LuminoGraph II.

### RNA sequencing and analysis

Total RNA was extracted from the collected tissues using TRIzol reagent according to the manufacturer’s protocol. The RNA concentration was measured using a NanoDrop spectrophotometer. The RNA sequencing (RNA-seq) libraries were prepared using a TruSeq Stranded mRNA Sample Prep Kit. Sequencing was performed using the Illumina NovaSeq 6000 platform. The data analysis was performed as previously described^17^. The differential gene expression was analyzed using the DESeq2 package (version 1.32.0). The data visualization was performed using the pheatmap, enhanced volcano and ggplot2 packages in R (version 4.4.1). The differentially expressed gene (DEG) results for each group comparison were evaluated using adjusted *P* values calculated using the the Benjamini–Hochberg method. All the data are presented as the mean ± standard error of the mean.

### Serotonin measurement

The sample preparation and measurements were performed as previously described^18^. The samples were homogenized in RIPA buffer, followed by centrifugation at 12,000*g* for 5 min at 4 °C. The supernatants were then mixed with methanol in a 1:1 ratio, centrifuged at 12,000*g* for 15 min at 4 °C and immediately frozen at −80 °C for subsequent analysis. The serotonin levels were measured using liquid chromatography with tandem mass spectrometry^19^.

### Hyperinsulinemic–euglycemic clamp test

A hyperinsulinemic–euglycemic clamp test was conducted to assess insulin sensitivity in the entire body and peripheral tissues. A total of 4 days before the experiment, a catheter was placed in the right internal jugular vein of each mouse. Following a 16-h overnight fast, the catheter was connected to a microdialysis pump (CMA Microdialysis AB) via a Y-shaped connector to infuse insulin (Lilly) and glucose. During the 2-h clamp study, insulin was infused at a rate of 24 pmol kg^−1^ min^−1^, and a 20% glucose solution was administered to maintain plasma glucose levels at approximately 6.5 mM. The blood samples were collected from the tail veins of each mouse. The insulin-stimulated whole-body glucose turnover rate was estimated via continuous infusion of [3–^3^H] glucose (0.1 μCi min^−1^, PerkinElmer). The glucose uptake in skeletal muscle was measured using a bolus injection of 2-deoxy-d-[1–^14^ C] glucose (10 μCi, PerkinElmer). The plasma glucose levels were assessed using a glucose analyzer (Analox), whereas plasma insulin concentrations were determined using an enzyme-linked immunosorbent assay (Merck). The hepatic glucose production during insulin infusion was calculated by subtracting the steady-state glucose infusion rate from the whole-body glucose turnover rate. The glucose uptake in skeletal muscle was determined from plasma 2-[^14^ C] DG profile analyzed with MLAB software (Civilized Software).

### ECAR assay

C2C12 cells were plated on an XF Pro M Cell Culture Microplate, following manufacturer protocols for use with the XF Pro analyzer. The cells were seeded at a concentration of 1000–5000 cells per well in 80 µl DMEM. When the cells reached 80% confluence, the medium was replaced with differentiation medium. The assay medium consisted of pH 7.4 XF DMEM assay medium supplemented with 1 M XF glucose, 200 mM XF glutamine and 100 mM XF pyruvate, all maintained at 37 °C. The XF assays were performed using serial injections of 0.5 µM rotenone plus antimycin A, followed by 50 mM 2-deoxyglucose, with 12 cycles of measurement (3 min each) and 3 min mixing intervals. Before the start of the assay, the cell plates were incubated in a non-CO_2_ incubator at 37 °C for 0.5–1 h for delayed measurement experiments. The extracellular acidification rates (ECARs) were measured using the Seahorse XF Glycolytic Rate Assay (Agilent 103344-100), and the values were normalized to the protein content.

### Histological analysis

For hematoxylin and eosin staining, the tissues were collected and fixed in 4% paraformaldehyde. After fixation, paraffin embedding, sectioning and hematoxylin and eosin staining were performed. The stained slides were imaged using an Olympus VS200 slide scanner. The myofiber size was analyzed using ImageJ and CellProfiler software. For the immunofluorescence analysis, the tissue sections were permeabilized on slides with a buffer containing saponin (Invitrogen) for 10 min at room temperature. The slides were then incubated in PBS with 3% (w/v) BSA for 1 h at room temperature, followed by incubation with anti-Glut4 antibody (1:300 dilution) at 4 °C overnight. After three 10-min washing with PBS, the slides were incubated with Alexa Fluor 594-conjugated goat anti-mouse secondary antibody for 2 h at room temperature in the dark. Finally, Fluoroshield with 4′,6-diamidino-2-phenylindole (DAPI) (F6057, Sigma) was used for nuclear staining. The cells were visualized using an Olympus FV3000RS confocal microscope.

For electron microscopic imaging, the samples were fixed in a solution of 2% glutaraldehyde and 2% paraformaldehyde in 0.1 M phosphate buffer (pH 7.4) for 12 h and then washed in 0.1 M phosphate buffer. Postfixation was performed using 1% osmium tetroxide (OsO_4_) in 0.1 M phosphate buffer for 2 h. The samples were then dehydrated using an ascending ethanol series for 10 min at each concentration, followed by infiltration with propylene oxide for 10 min. Transmission electron microscopy sample preparation and imaging were performed at the Yonsei Advanced Imaging Center. The samples were embedded using a Poly/Bed 812 kit (Polysciences) and polymerized in an electron microscope furnace (TD-700, DOSAKA) at 65 °C for 12 h. The blocks were cut using a diamond knife on an ultramicrotome and sectioned into 200-nm slices, which were stained with toluidine blue for observation under a light microscope. The regions of interest were further sectioned at 80 nm, placed on copper grids and double-stained with 3% uranyl acetate for 30 min followed by 3% lead citrate for 7 min. The imaging was performed using a transmission electron microscope (JEM-1011, JEOL) equipped with a MegaView III CCD camera (Soft Imaging System) at an acceleration voltage of 80 kV.

### Statistical analysis

The sample size, number of replicates for each experimental group and number of independent experiments are shown in the corresponding figure legends. The data are represented as the mean ± standard error of the mean. The multigroup comparisons were performed using one-way or two-way analysis of variance with Bonferroni post hoc tests in GraphPad Prism version 10.3 (GraphPad Software, Inc.) or R 4.4.1, with *P* values < 0.05 considered statistically significant.

## Results

### Inhibition of 5-HT synthesis improves glucose uptake signaling in C2C12 myotubes

**Fig. 1:**
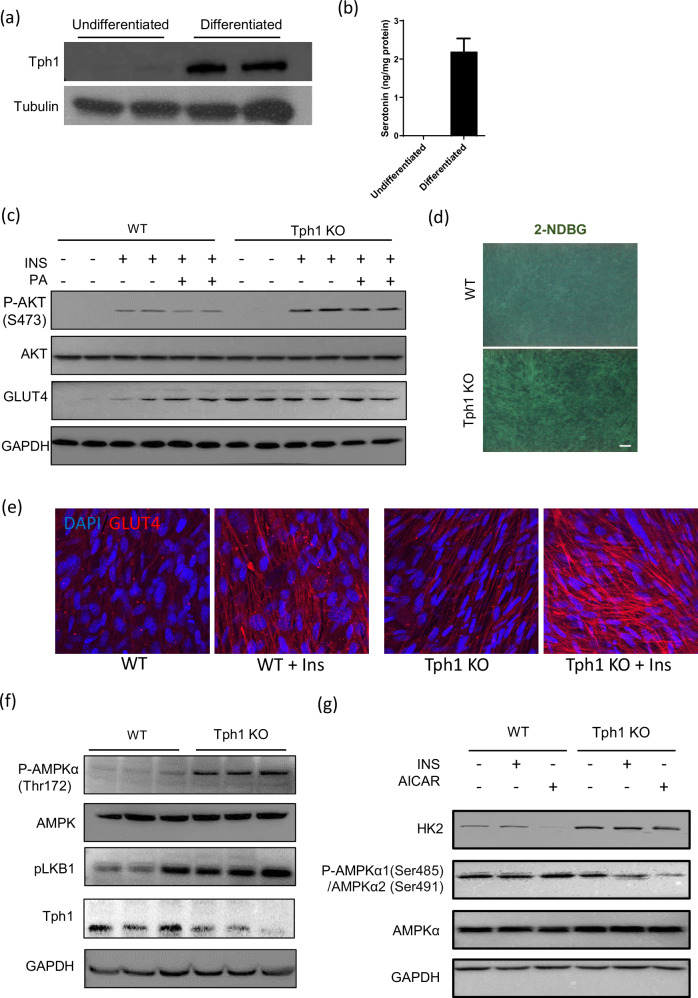
Effects of inhibiting serotonin on C2C12 myotubes. **a** The western blot images of Tph1 expression in undifferentiated and differentiated C2C12 cells, with tubulin as the loading control. **b** The serotonin levels in undifferentiated and differentiated C2C12 cells. **c** Western blot images showing the levels of AKT, phosphorylated AKT^S473^, GLUT4 and GAPDH in WT and *Tph1*-KO C2C12 cells with or without insulin stimulation for 15 min after 24 h of 0.5 mM palmitate treatment. **d** The representative images of 2-NBDG glucose uptake in WT and *Tph1*-KO cells. Scale bar, 100 µm. **e** Representative immunofluorescence images of GLUT4 expression in C2C12 myotubes treated with or without 100 nM insulin for 30 min. **f** The western blot images of phosphorylated AMPKα^Thr172^, AMPK, phosphorylated LKB1 and Tph1 in WT and *Tph1*-KO cells. **g** The western blot images of hexokinase (HK), phosphorylated AMPKα1^Ser485/^α2^Ser491^ and AMPKα in WT and *Tph1*-KO cells after treatment with either 100 nM insulin or 2 mM AICAR.

To determine whether skeletal muscle cells can produce 5-HT, we evaluated the expression of tryptophan hydroxylase 1 (Tph1)^14^, the rate-limiting enzyme in 5-HT synthesis. A western blot analysis was used to detect Tph1 expression in differentiated C2C12 myotubes (Fig. 1a). Reverse transcription PCR (RT–PCR) was used to identify Htrs in C2C12 myotubes (Supplementary Fig. 1a). Intracellular 5-HT levels were also detected in differentiated myotubes (Fig. 1b). These findings suggest that 5-HT plays a more critical role in modulating the physiological functions of differentiated muscle fibers than in early myogenic processes or muscle stem cell activity. We then generated *Tph1*-KO C2C12 myotubes using a CRISPR–Cas9 system to evaluate the role of 5-HT in glucose metabolism. Depleting 5-HT in C2C12 myotubes led to increased insulin-stimulated AKT phosphorylation, even in palmitate C2C12 cells (Fig. 1c).

Glut4 protein expression, essential for insulin-mediated glucose transport in skeletal muscle^20^, was increased in *Tph1*-KO myotubes compared with wild-type (WT) myotubes, and this increase was accompanied by an increase in glucose uptake (Fig. 1c, d). In addition, immunofluorescence staining revealed elevated Glut4 levels in insulin-stimulated *Tph1*-KO myotubes versus WT cells (Fig. 1e).

AMPK (5′-adenosine monophosphate-activated protein kinase) is a key cellular energy sensor^21^ that plays a critical role in the regulation of skeletal muscle metabolism, fincluding glucose uptake^22^ and fatty acid oxidation^23^. *Tph1*-KO myotubes exhibited increased Lkb1 and AMPKα^Thr172^ phosphorylation and reduced AMPKα1^Ser485^/α2^Ser491^ phosphorylation, indicating enhanced Lkb1/AMPK signaling (Fig. 1f, g)^24,25^. In addition, hexokinase 2 (Hk2) levels were higher in *Tph1*-KO myotubes than in WT myotubes (Fig. 1g).

### Inhibition of 5-HT synthesis in skeletal muscle protects against HFD-induced insulin resistance

**Fig. 2:**
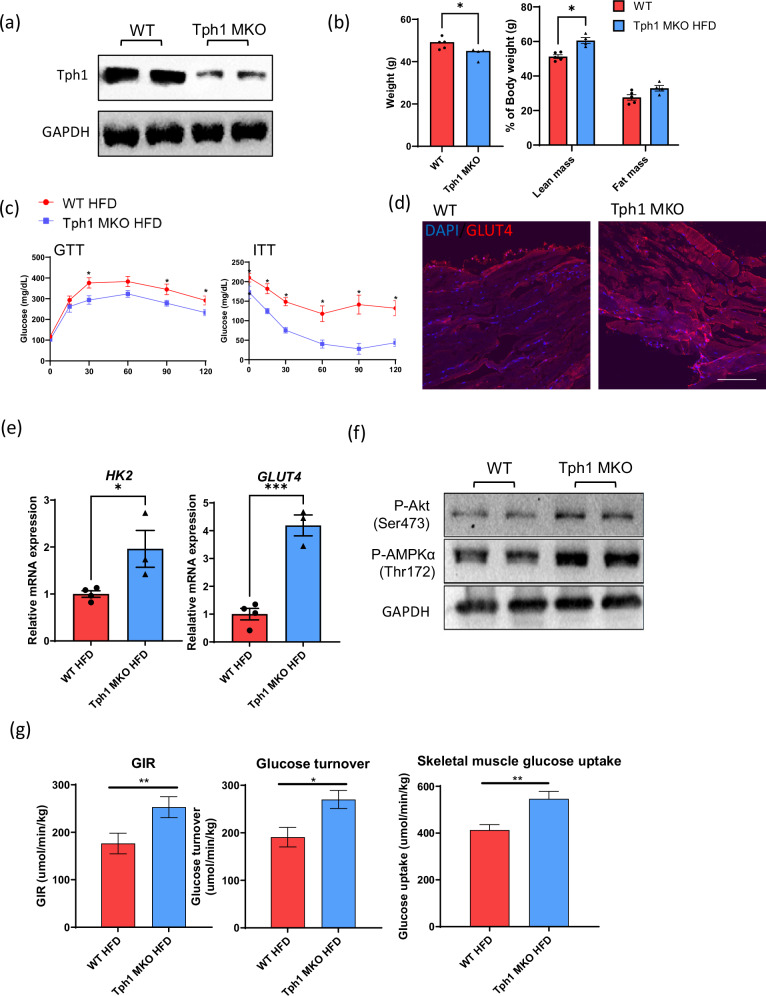
*Tph1* KO in skeletal muscle protects against HFD-induced obesity and insulin resistance. **a** The Tph1 expression in quadriceps muscle of WT and *Tph1* muscle-specific-KO (*Tph1* MKO) mice. **b** Body weight and body composition analysis (*n* = 4 or 5 per group) measured via dual energy X-ray absorptiometry after 12 weeks of HFD feeding. **c** The results of the GTT and ITT after 16 and 6 h of fasting, respectively (*n* = 4–6 per group). **d** Immunofluorescence staining of GLUT4 (red) in quadriceps muscle from WT and *Tph1* MKO skeletal muscle. The nuclei were counterstained with DAPI (blue). Scale bar, 100 µm. **e** The relative mRNA expression of Hk2 and GLUT4 in quadriceps muscle from mice fed a HFD, assessed via qRT–PCR. **f** Western blot images showing phosphorylated AKT and phosphorylated AMPKα^Thr172^ from WT and *Tph1* MKO skeletal muscle after HFD feeding. **g** The measurement of insulin levels, glucose turnover and skeletal muscle glucose uptake in WT and *Tph1* MKO mice during clamp studies. GIR, glucose infusion rate. *P < 0.05, **P < 0.01.

To evaluate the role of 5-HT in skeletal muscle in vivo, we generated skeletal muscle-specific *Tph1*-KO (*Tph1* MKO) mice (Fig. 2a and Supplementary Fig. 1b) and evaluated their phenotypes in a diet-induced obesity model. Both WT and *Tph1* MKO mice exhibited similar body weight, glucose tolerance and insulin resistance (Supplementary Fig. 1c–e). However, after 12 weeks of HFD feeding, *Tph1* MKO mice exhibited reduced body weight, increased lean mass and improved glucose tolerance and insulin sensitivity compared with WT mice (Fig. 2b, c).

Immunofluorescence staining revealed increased Glut4 expression in the skeletal muscle of *Tph1*-KO mice compared with that of WT mice (Fig. 2d). In addition, the relative expression levels of *Hk2* and *Glut4* were significantly higher in *Tph1*-KO mice (Fig. 2e). The protein analysis further revealed an increase in Glut4 expression as well as increased phosphorylation of AKT and AMPKα^Thr172^ in *Tph1*-KO mice compared with WT mice (Fig. 2f and Supplementary Fig. 1f). The hyperinsulinemic–euglycemic clamp study also showed improved insulin sensitivity and glucose uptake in the skeletal muscle of *Tph1*-KO mice (Fig. 2g).

### Inhibition of 5-HT synthesis in skeletal muscle protects against HFD-induced myosteatosis

**Fig. 3:**
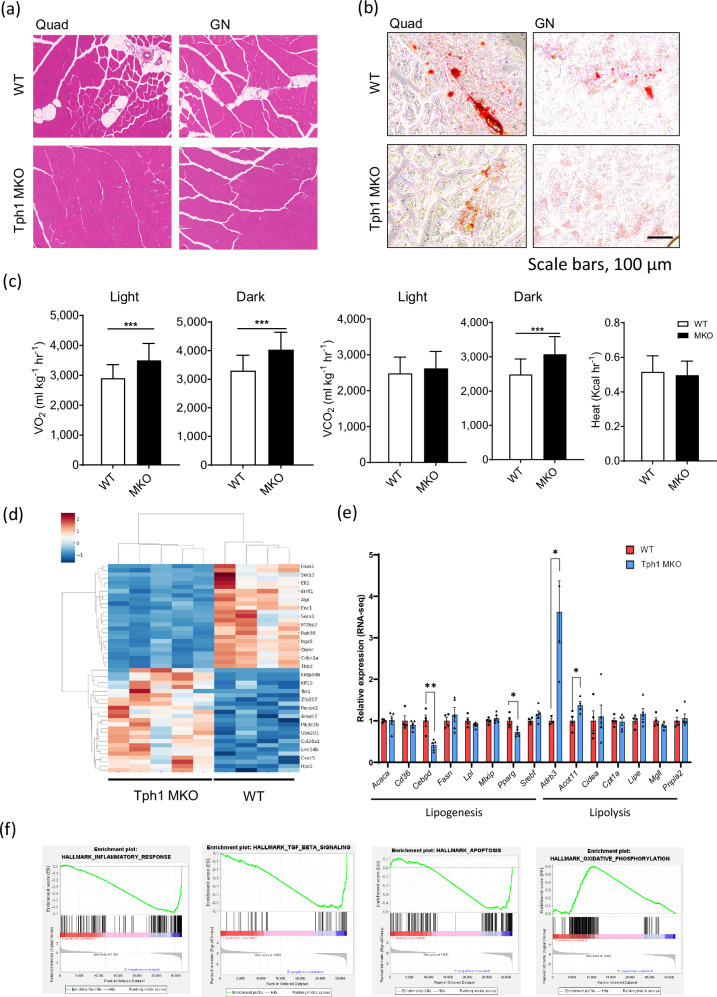
Effect of *Tph1* muscle KO on energy metabolism. **a** Hematoxylin and eosin (H&E) staining of quadriceps (Quad) and gastrocnemius (GN) muscles from WT and *Tph1* MKO mice after 12 weeks of HFD feeding. **b** Oil Red O staining of skeletal muscle sections. Scale bar, 100 µm. **c** The metabolic rates of WT and *Tph1* MKO mice under light and dark cycles after 12 weeks of HFD feeding. **d** A heat map showing the DEGs identified via bulk RNA-seq analysis of skeletal muscle from WT and *Tph1*-KO mice. **e** The relative expression of genes involved in lipogenesis and lipolysis from skeletal muscle transcriptome data of WT and *Tph1* MKO mice. **f** The enrichment plots for the inflammatory response pathway, TGF-beta signaling pathway, apoptosis pathway and oxidative phosphorylation pathway. *P < 0.05, **P < 0.01, *** P < 0.001.

Excessive fat accumulation in skeletal muscle, termed myosteatosis, has been positively correlated with insulin resistance associated with obesity^26,27^. After 12 weeks of HFD feeding, histological analysis revealed a significant reduction in lipid deposition in the skeletal muscle of *Tph1*-KO mice compared with that in WT controls (Fig. 3a, b). In addition, metabolic cage assays revealed increased oxygen consumption in *Tph1*-KO mice, suggesting increased energy expenditure and metabolic activity compared with WT mice (Fig. 3c).

To obtain a comprehensive understanding of the effects of 5-HT depletion on skeletal muscles, we conducted RNA sequencing (RNA-seq) of skeletal muscle tissues from HFD-fed *Tph1*-KO and WT mice. Figure 3d shows a heat map illustrating the DEGs between the *Tph1*-KO and WT groups, highlighting the distinct gene expression profiles between the two conditions. The top 20 upregulated and top 20 downregulated DEGs were ranked according to log_2_ fold change, with an adjusted *P* value <0.05 (Supplementary Fig. 2a). The RNA-seq data revealed decreased expression of lipogenic genes, including CCAAT/enhancer-binding protein delta (*Cebpd*) and peroxisome proliferator-activated receptor gamma (*Pparg*) and increased expression of lipolytic genes, including adrenoceptor beta 3 (*Adrb3*) and acyl-CoA thioesterase 11 (*Acot11*) (Fig. 3e). We then performed a gene set enrichment analysis (GSEA) of the DEGs using the RNA-seq data. The results negative enrichment for inflammation-related genes and positive enrichment for genes involved in oxidative phosphorylation (Fig. 3f and Supplementary Fig. 2b).

To further investigate the biological functions of the DEGs, we performed gene ontology (GO) functional classification analysis (Supplementary Fig. 2c). In the biological process (BP) category, the top three significantly enriched terms were ‘biological regulation’, ‘metabolic process’ and ‘response to stimuli’. For the cellular component category, the most enriched terms were ‘membrane’, ‘nucleus’ and ‘protein-containing complexes’. In the molecular function category, the most enriched terms were ‘protein binding’, ‘ion binding’ and ‘nucleic acid binding’.

### 5-HT regulates glucose metabolism through Htr2b in C2C12 myotubes

**Fig. 4:**
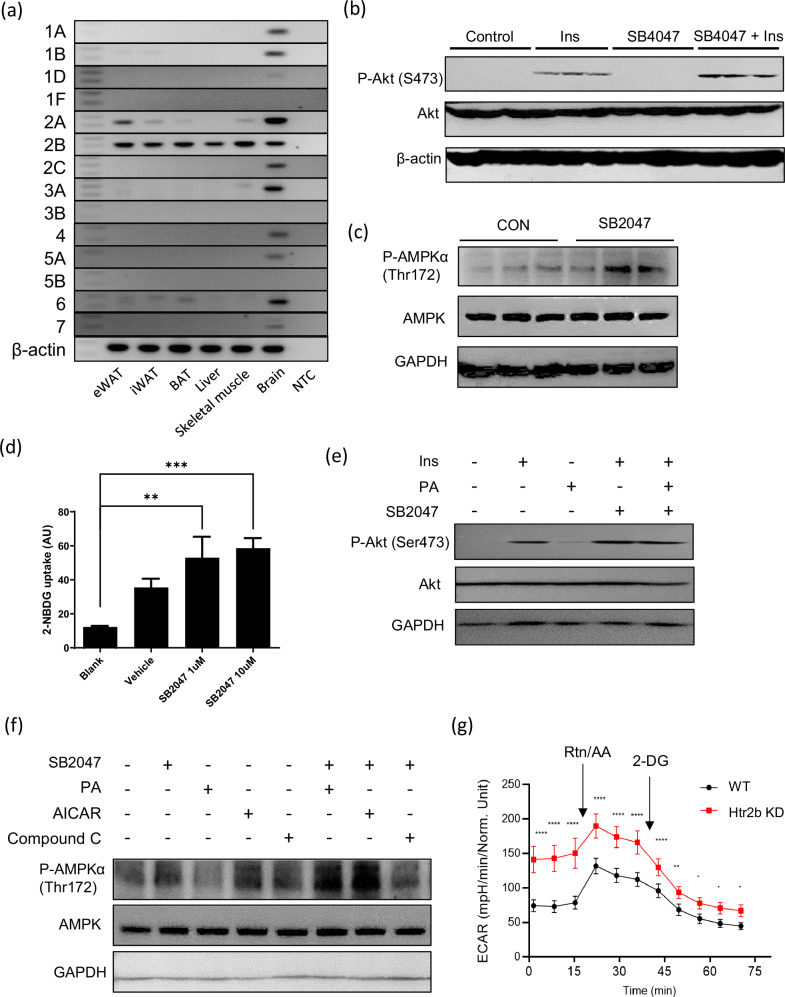
Effect of inhibiting Htr2b signaling on C2C12 myotubes. **a** mRNA detection of 5-HT receptors (Htrs) in brain and peripheral tissues using RT–PCR. eWAT, epididymal white adipose tissue; iWAT, inguinal white adipose tissue; BAT, brown adipose tissue; NTC, no template control. **b** Western blot images showing the effect of SB204741 (an Htr2B inhibitor, SB2047, 50 nM) on phosphorylated AKT^Ser473^ and total AKT levels in C2C12 myotubes with or without insulin treatment (100 nm for 30 min). **c** Western blot images showing the effect of SB2047 on phosphorylated AMPKα^Thr172^ and total AMPK levels in C2C12 myotubes. **d** A quantification of 2-NBDG glucose uptake in C2C12 myotubes treated with SB2047. **e** Western blot images showing the effect of SB2047 on phosphorylated AKT^Ser473^ and total AKT levels in C2C12 myotubes with palmitate (PA) (0.5 mM for 48 h). **f** Western blot images showing phosphorylated AMPKα^Thr172^ in C2C12 myotubes with PA. CC, Compound C. **g** The ECAR was measured in WT and *Htr2b* knockdown C2C12 myotubes after sequential injections of compounds rotenone and antimycin cocktail (Rot/AA) and 2-deoxy-d-glucose (2-DG).

The physiological effects of 5-HT are mediated by various Htrs involved in different biological processes. To determine the Htr subtype that mediates the role of 5-HT in the development of insulin resistance in skeletal muscles, we analyzed the expression of several Htr genes in mouse tissues. Notably, the expression of *Htr2b* was higher than that of other *Htrs* in the skeletal muscle, suggesting a possible link between Htr2b and skeletal muscle energy metabolism (Fig. 4a). When we blocked Htr2b signaling with SB204741, an Htr2b inhibitor^28^ in C2C12 myotubes, we observed a marked increase in insulin-stimulated AKT phosphorylation (Fig. 4b). The phosphorylation of AMPKα^Thr172^ was also significantly increased (Fig. 4c). Moreover, SB204741 treatment increased glucose uptake in C2C12 myotubes (Fig. 4d). Knockdown of *Htr2b* in C2C12 myotubes also increased insulin-stimulated AKT phosphorylation and elevated Glut4 expression in palmitate-treated cells (Supplementary Fig. 3a,b).

In addition, SB204741 treatment effectively reversed palmitate-induced insulin resistance (Fig. 4e) and increased AMPK α^Thr172^ phosphorylation in C2C12 myotubes (Fig. 4f), indicative of enhanced metabolic function. To further investigate these effects, we measured the ECAR using a Seahorse Extracellular Flux Analyzer. The results showed that *Htr2b* knockdown C2C12 myotubes exhibited a significantly higher ECAR than WT cells (Fig. 4g), suggesting increased glycolytic activity in the absence of Htr2b signaling. These results highlight the central role of 5-HT-Htr2b signaling in the regulation of glucose uptake and energy metabolism in C2C12 myotubes.

### Inhibition of Htr2b signaling in skeletal muscle protects against HFD-induced myosteatosis

**Fig. 5:**
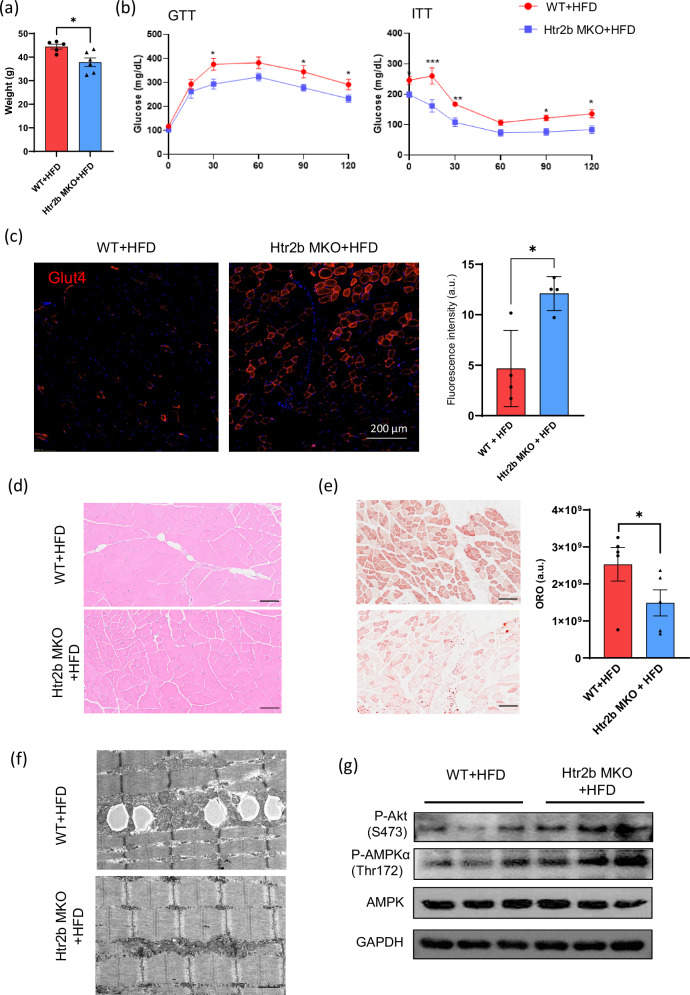
*Htr2b* KO in skeletal muscle protects against HFD-induced obesity and insulin resistance. **a** The body weight of WT and *HTR2b* muscle-specific-KO (*Htr2b* MKO) mice after 12 weeks of HFD feeding. **b** GTT and ITT in WT and *Htr2b* MKO mice. **c** Immunofluorescence staining of Glut4 (red) in quadriceps muscle from WT and *HTR2b* MKO mice. The nuclei were counterstained with DAPI (blue). Scale bar, 100 µm. **d** Hematoxylin and eosin staining of quadriceps muscle from WT and *HTR2b* MKO mice after 12 weeks of HFD feeding. **e** Oil Red O staining of skeletal muscle sections. Scale bar, 100 µm. **f** Representative electron micrograph of skeletal muscle (quadriceps) from WT and *Htr2b* MKO mice. Scale bar, 10 µm. **g** Western blot images showing phosphorylated AKT^Ser473^, phosphorylated AMPKα^Thr172^ and total AMPK levels in skeletal muscle from WT and *Htr2b* MKO mice.

To explore the role of Htr2b in the development of insulin resistance and myosteatosis, we generated muscle-specific *HTR2b*-KO (*HTR2b* MKO) mice. After 12 weeks of HFD feeding, *Htr2b* MKO mice showed reduced body weight gain and improved glucose tolerance compared with WT mice (Figs. 5a, b). Immunofluorescence staining further revealed increased expression of Glut4 in the skeletal muscle of *HTR2b* MKO mice compared with WT mice (Fig. 5d and Supplementary Fig. 3c,d), suggesting enhanced glucose uptake in KO mice. Interestingly, quantitative analysis of muscle glycogen content showed no significant differences between *Htr2b-*KO (*Htr2b* MKO) and WT mice (Supplementary Fig. 3e). These observations suggest that despite increased Glut4 expression and improved insulin sensitivity, the additional glucose imported into muscle cells is predominantly directed toward glycolysis rather than glycogen synthesis.

Histological analysis revealed a significant reduction in lipid deposition in the skeletal muscle of *Htr2b*-KO mice compared with that in WT mice (Fig. 5d, e). It also revealed that liver and adipose tissue in *Htr2b*-KO mice did not show significant differences compared with WT controls after HFD (Supplementary Fig. 4). HFD-induced muscle insulin resistance typically results in a gradual increase in mitochondrial content as an adaptive response to elevated free fatty acid levels^29^. However, in this study, electron microscopy revealed that skeletal muscle from HFD-fed *Htr2b*-KO mice exhibited smaller lipid droplets and reduced mitochondrial size compared with HFD-fed WT mice (Fig. 5f). In addition, immunoblot assays revealed increased phosphorylation of AKT and AMPKα^Thr172^ in *Htr2b*-KO mice compared with WT mice (Fig. 5g). These findings suggest that alterations in lipid storage and mitochondrial morphology, coupled with enhanced AKT and AMPK signaling, contributed to the improved metabolic outcomes observed in *Htr2b*-KO mice under HFD conditions.

### Inhibition of Htr2b signaling in skeletal muscle leads to transcriptional changes in energy metabolism

**Fig. 6:**
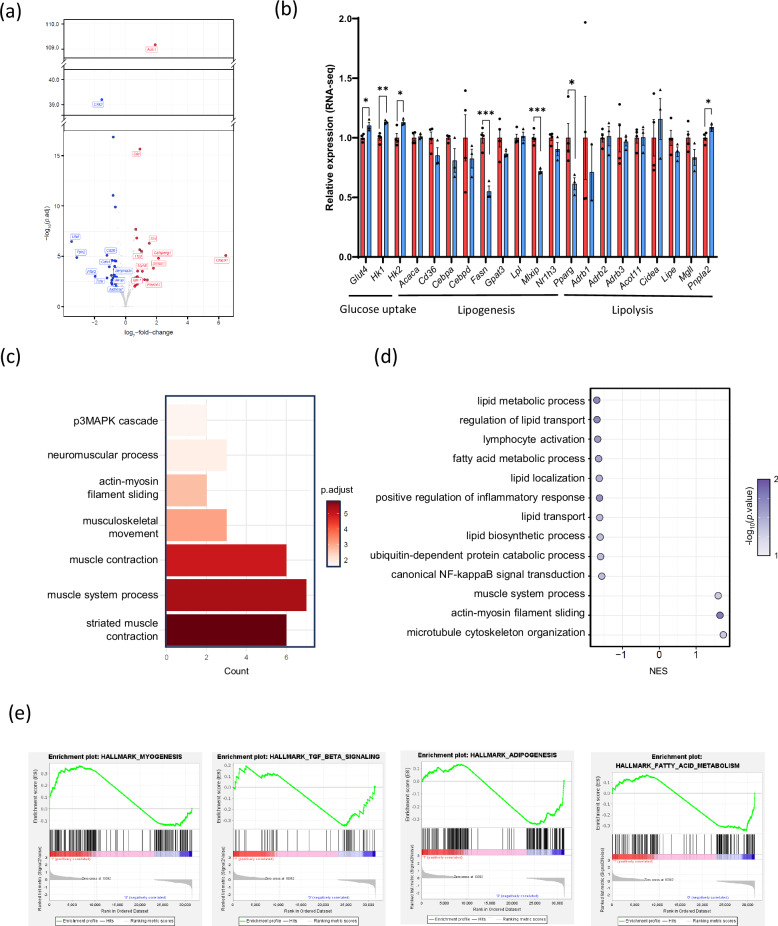
Transcriptome analysis of skeletal muscle of WT and *Htr2b* MKO mice. **a** A heat map showing DEGs identified via bulk RNA-seq analysis of skeletal muscle from WT and *HTR2b*-KO mice. **b** The relative expression of genes involved in glucose uptake, lipogenesis and lipolysis from skeletal muscle transcriptome data of WT and *Htr2b* MKO mice. **c** **d** A pathway enrichment analysis of DEGs, GO (**c**) and GSEA (**d**). **e** Enrichment plots for the myogenesis pathway, TGF-beta signaling pathway, adipogenesis pathway and fatty acid metabolism pathway. *P < 0.05, **P < 0.01, ***P < 0.001.

To investigate the role of Htr2b in skeletal muscle energy metabolism, we performed an RNA-seq analysis of skeletal muscle tissues from HFD-fed *Htr2b*-KO and WT mice. Figure 6a shows a volcano plot illustrating the DEGs between the *Htr2b*-KO and WT mice. The detailed list of the top ten upregulated genes and the top ten downregulated genes is shown in Supplementary Table 1. Glucose metabolism-related genes were upregulated, while lipogenic genes were downregulated in *Htr2b* KO compared with those in WT mice (Fig. 6b and Supplementary Fig. 5a, b).

We then performed comprehensive pathway analyses using GO and GSEA to further interrogate the transcriptomic changes in skeletal muscle. GO analysis revealed significant upregulation of pathways associated with muscle function, including ‘musculoskeletal development’ and ‘striated muscle contraction’ (Fig. 6c). GSEA also revealed that pathways related to lipid metabolism, such as ‘lipid metabolic process’, ‘fatty acid metabolic process’ and ‘lipid biosynthetic process’ were negatively enriched (Fig. 6d). Furthermore, the GSEA plot showed positive enrichment of gene sets associated with ‘myogenesis’, while negative enrichment was observed for gene sets associated with ‘adipogenesis’, ‘fatty acid metabolism’ and ‘TGF-beta signaling’ (Fig. 6e). Complementary GO functional classification analysis was also performed (Supplementary Fig. 5c). In the BP category, the top three significantly enriched terms were ‘biological regulation’, ‘metabolic process’ and ‘response to stimuli’. For the cellular component category, the top enriched terms were ‘membrane’, ‘nucleus’ and ‘protein-containing complexes’. In the molecular function category, the top enriched terms were ‘protein binding’, ‘ion binding’ and ‘hydrolase activity’.

## Discussion

In this study, we demonstrate that inhibiting 5-HT signaling through Htr2b in skeletal muscle improves insulin sensitivity in HFD-induced obese mice. By specifically inhibiting 5-HT synthesis in skeletal muscles using *Tph1*-KO mice, we observed a marked improvement in glucose tolerance (Fig. 2c) and reduced myosteatosis in obese mice (Fig. 3a, b). This improvement was accompanied by increased expression of Glut4 (Fig. 2d, f) and AKT phosphorylation, suggesting enhanced glucose uptake and metabolism. Furthermore, 5-HT synthesis inhibition increased AMPK phosphorylation in both C2C12 myotubes and skeletal muscle tissues (Figs. 1f, g and 2f), which are essential regulators of glycolysis^30^ and fatty acid oxidation^23^.

Pharmacological inhibition of Htr2b signaling by SB204741 underscores the critical role of Htr2b in the regulation of insulin signaling and glucose metabolism. In C2C12 myotubes, Htr2b inhibition resulted in increased phosphorylation of both AKT and AMPK (Fig. 4b, c) and increased glycolytic activity (Fig. 4g). In vivo muscle-specific KO of *Htr2b* in HFD-fed mouse models further underscored the therapeutic potential of targeting this pathway. *Htr2b* MKO mice exhibited improved insulin sensitivity (Fig. 5b), Glut4 expression (Fig. 5c and Supplementary Fig. 3c, d) and a marked reduction in skeletal muscle lipid accumulation (Fig. 5d, e). Excessive lipid deposition in the skeletal muscle, a condition known as myosteatosis, is strongly associated with insulin resistance and metabolic dysfunction^31^. Our results suggest that Htr2b inhibition mitigates these detrimental effects, potentially reversing insulin resistance associated with muscle lipid overload. Furthermore, electron microscopy revealed a decrease in lipid content and an increase in mitochondrial density in the skeletal muscle of *Htr2b* MKO mice compared with that of WT mice (Fig. 5f), suggesting that Htr2B inhibition may protect mitochondria from lipotoxic stress^32^. Moreover, RNA-seq analysis of both *Tph1* MKO and *Htr2b* MKO mice revealed transcriptional shifts toward upregulated glycolytic and lipolytic pathways and downregulated lipogenic genes (Figs. 3 and 6), indicating an overall shift in skeletal muscle energy metabolism toward a more metabolically favorable state.

In this study, we demonstrate that the inhibition of 5-HT-Htr2b signaling resulted in enhanced insulin-stimulated AKT and AMPK signaling in skeletal muscle, suggesting that this pathway may be a critical mechanism underlying the beneficial metabolic effects observed with 5-HT-Htr2b inhibition. The role of AKT in regulating glucose metabolism is well established; for example, it facilitates glucose uptake by increasing the transcription and translocation of glucose transporters while also promoting glycolysis^33,34^. Thus, enhanced AKT signaling leads to increased glucose uptake in the skeletal muscle, improving cellular energy availability and potentially enhancing insulin sensitivity^35^. Similarly, AMPK is a key regulator of energy homeostasis^36^ and is known to promote glucose uptake^37,38^, fatty acid oxidation^36^ and mitochondrial biogenesis in skeletal muscle^39^. AMPK activation also decreases lipid accumulation in skeletal muscle cells^40^. Conversely, reduced AMPK activity has been associated with increased lipid accumulation and insulin resistance, further highlighting the importance of maintaining AMPK activity for maintaining metabolic health^41^.

In addition, the inhibition of 5-HT-Htr2b signaling also resulted in enhanced lipolysis accompanied by increased activation of both AKT and AMPK. While classical insulin signaling via AKT normally suppresses lipolysis through phosphodiesterase-mediated cAMP degradation, this antilipolytic effect may be diminished under metabolic stress^42^, allowing robust AMPK activation to drive lipolysis. Further studies are needed to elucidate the precise crosstalk between these pathways in regulating lipid metabolism under such conditions.

Our findings suggest that increased activation of both AKT and AMPK may represent a key mechanistic pathway by which 5-HT-Htr2b inhibition improves metabolic function in skeletal muscle. In parallel, pharmacological inhibition of Htr2b in C2C12 myotubes not only restored insulin sensitivity but also shifted the transcriptional profile toward upregulated glycolytic and lipolytic pathways and enriched gene sets associated with myogenesis. These findings suggest that muscle-derived 5-HT exerts autocrine/paracrine effects that modulate key signaling cascades and gene expression programs critical for both energy homeostasis and muscle differentiation, extending the traditional view of 5-HT as a predominantly gut-derived hormone and highlighting its potential as a therapeutic target in metabolic and muscle disorders.

Our study has several limitations that warrant further investigation. First, we were unable to quantify local 5-HT levels in skeletal muscle tissue or assess its potential interactions with other serotonin receptor subtypes, largely due to the technical challenges associated with detecting extremely low endogenous concentrations and the rapid degradation of 5-HT by monoamine oxidase. Future studies using more sensitive analytical methods are essential to elucidate the precise role and regulation of 5-HT in muscle physiology and to understand its interplay with other serotonergic pathways. Second, although our study provides compelling evidence for the role of Htr2b in skeletal muscle metabolism, the precise mechanism by which 5-HT modulates AKT and AMPK expression through Htr2b remains unclear. The precise molecular mechanisms underlying the observed mitochondrial changes in skeletal muscle also remain to be fully elucidated. Further research is needed to determine how Htr2b signaling affects mitochondrial dynamics and other metabolic regulators in skeletal muscle. Finally, although we demonstrated the therapeutic potential of Htr2b inhibition in a mouse model, the translation of these findings into clinical applications requires further validation.

Our study identifies Htr2b as a novel regulator of skeletal muscle insulin sensitivity and highlighted the therapeutic potential of targeting skeletal muscle 5-HT signaling in the treatment of obesity-related metabolic diseases. These findings pave the way for the development of novel therapies targeting peripheral 5-HT pathways to combat insulin resistance and its associated complications.

## Supplementary information

Supplementary Information

## Data Availability

Sequencing data are accessible from the Gene Expression Omnibus database (GSE275857 and GSE279210).
