# Author Correction: Inhibition of serotonin-Htr2b signaling in skeletal muscle mitigates obesity-induced insulin resistance

**DOI:** 10.1038/s12276-025-01492-3

**Published:** 2025-06-30

**Authors:** Suhyeon Park, Hyeongseok Kim, Soyeon Shin, Yeongmin Kim, Yunwon Kang, Hye-Na Cha, So-Young Park, Sangkyu Park, Chang-Myung Oh

**Affiliations:** 1 Department of Biomedical Science and Engineering, Gwangju Institute of Science and Technology, Gwangju, Republic of Korea; 2 Department of Biochemistry, College of Medicine, Chungnam National University, Daejeon, Republic of Korea; 3 Department of Physiology, College of Medicine, Yeungnam University, Daegu, Republic of Korea; 4 Senotherapy-based Metabolic Disease Control Research Center, College of Medicine, Yeungnam University, Daegu, Republic of Korea; 5 Department of Biochemistry, Yonsei University Wonju College of Medicine, Wonju, Republic of Korea; 6 Organelle Medicine Research Center, Yonsei University Wonju College of Medicine, Wonju, Republic of Korea

**Keywords:** Metabolic syndrome, Transcriptomics

Correction to: *Experimental & Molecular Medicine* 10.1038/s12276-025-01460-x, published online 2 June 2025

After the online publication of this article, the authors noticed an error in the “Results” section.

The originally published version of the article incorrectly presented Fig. 5B. This figure corresponds to the results of the insulin tolerance test. The corrected version is provided below.
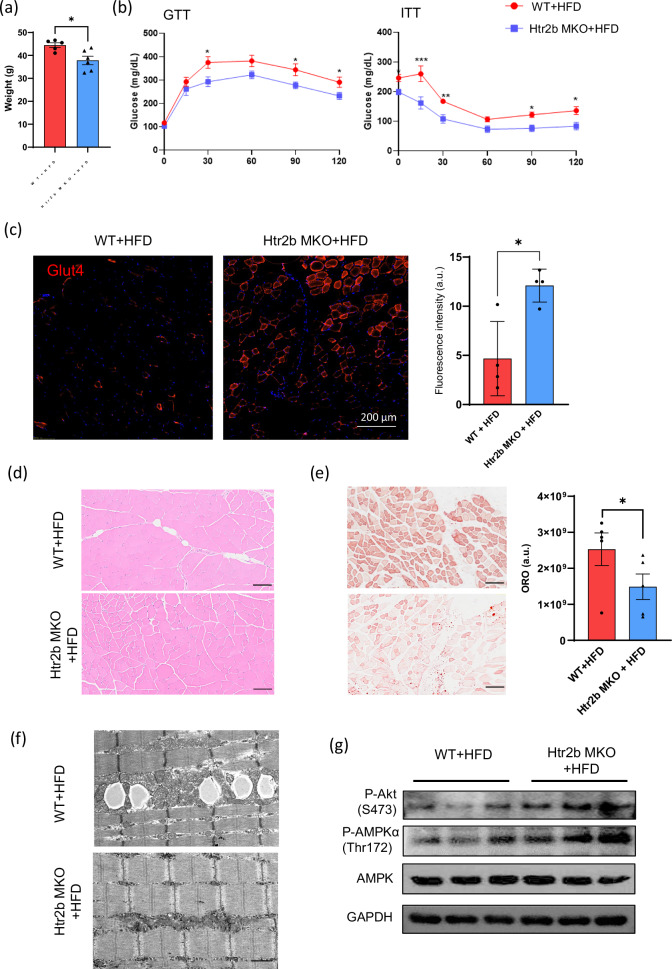


The authors apologize for any inconvenience caused.

The original article has been corrected.

