# Supplementary material for: Inhibition of serotonin-Htr2b signaling in skeletal muscle mitigates obesity-induced insulin resistance
Source: Exp Mol Med. 2025 Jun 2;57(6):1177–88. doi: 10.1038/s12276-025-01460-x (PMC12227683; doi:10.1038/s12276-025-01460-x)
Supplement: Supplementary file 1 — Supplementary Information [file 12276_2025_1460_MOESM1_ESM.pdf]

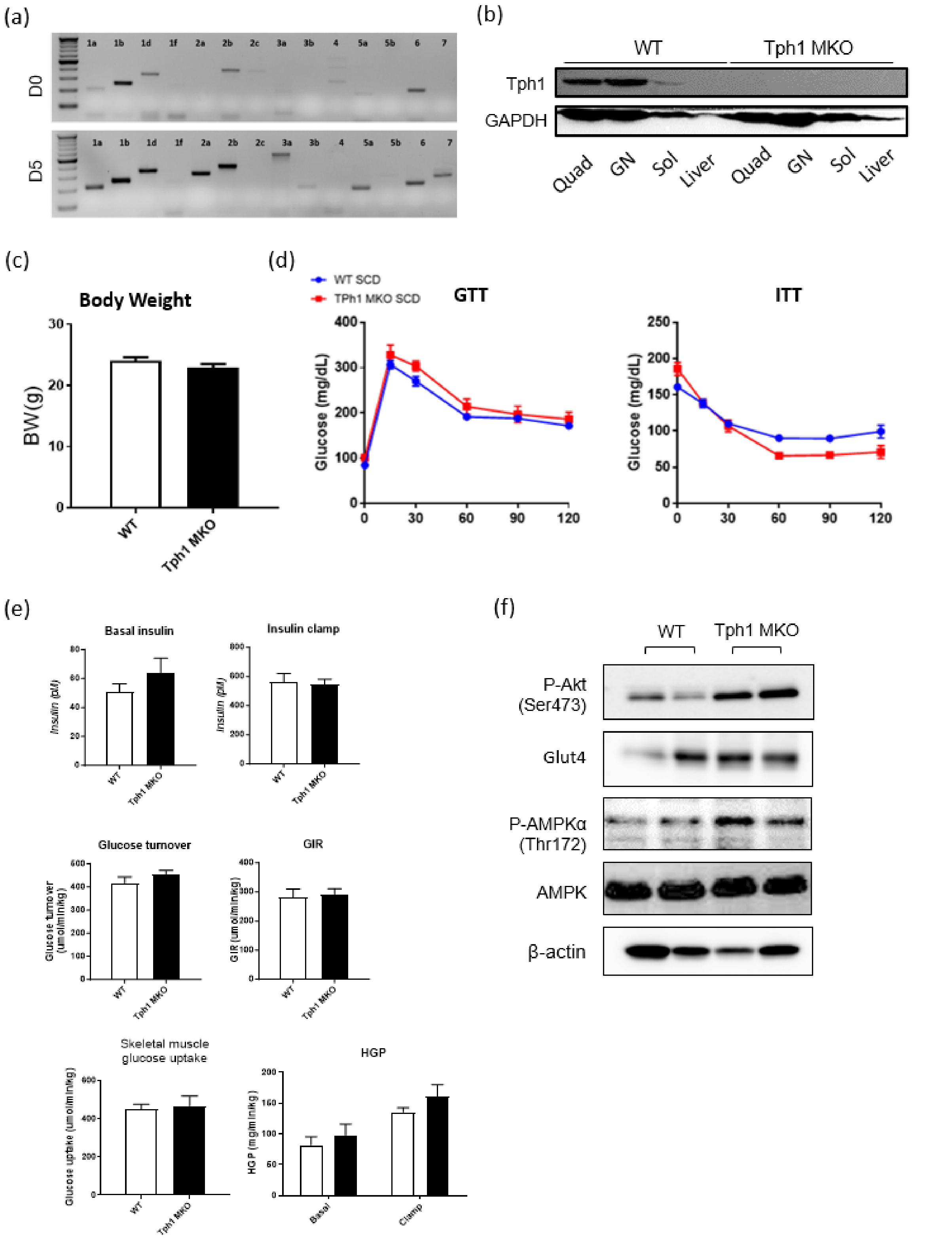

Supplementary Fig. 1. (a) Serotonin receptor mRNA expression in C2C12 myoblast (D0) and myotube (D5). (b) Tph1 expression in peripheral tissues of wild-type (WT) and *Tph1* muscle-specific KO (*Tph1* MKO) mice. (c) Body weight of WT and *Tph1* MKO mice at 8–10 weeks of age on a standard chow diet (SCD). (d) Glucose tolerance test (GTT) after 16 hours

fasting and insulin tolerance test (ITT) after 6 hours fasting (n=4-6/group). (e) Measurement of insulin levels, glucose turnover and skeletal muscle glucose uptake in WT and *Tph1* MKO mice during clamp studies. GIR, glucose infusion rate.(f) Western blot images showing phosphorylated AKT, GLUT4, phosphorylated AMPKα^{Thr172}, and AMPK.

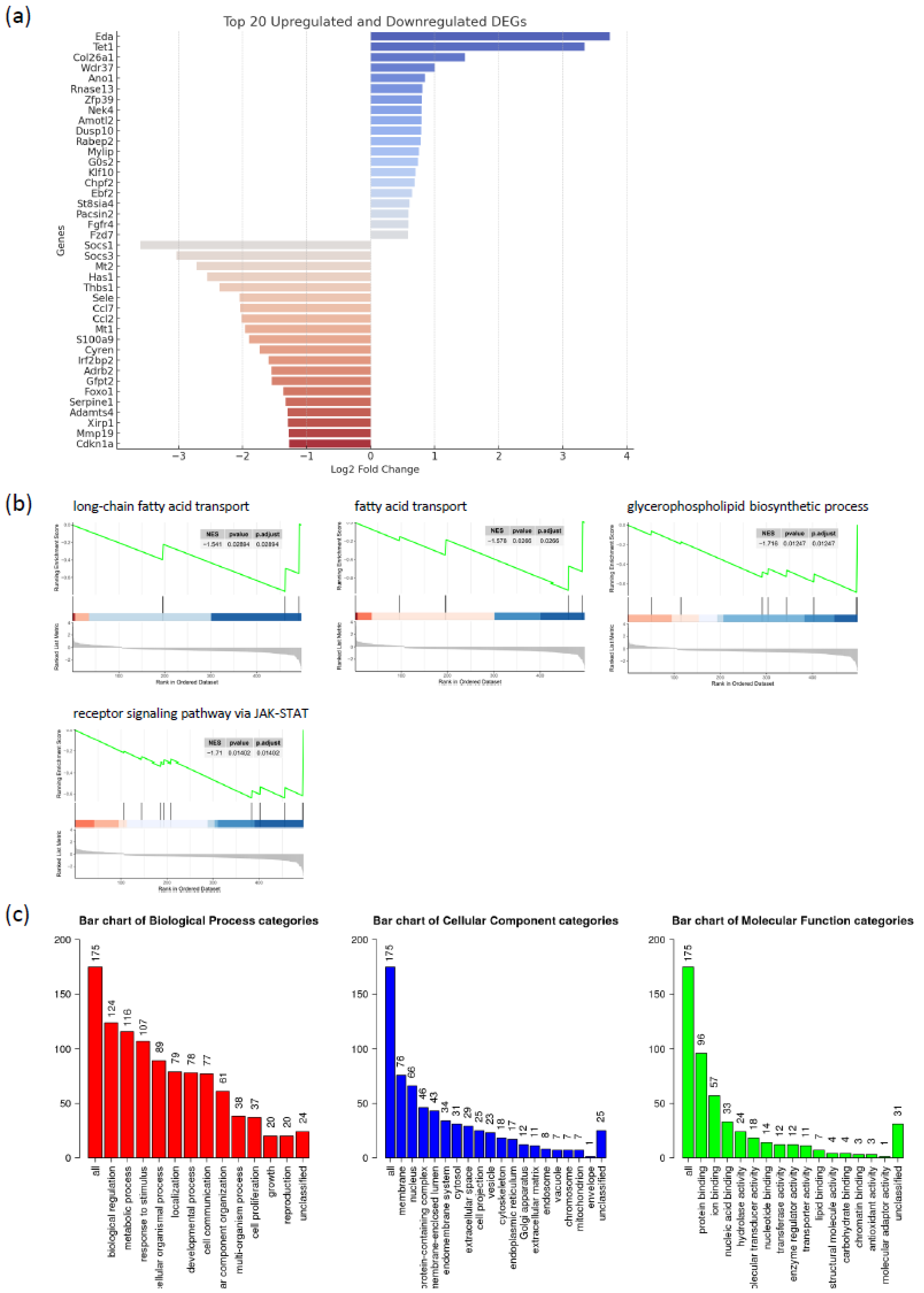

Supplementary Fig. 2. (a) Top 20 up- and downregulated differentially expressed genes (DEGs) in the skeletal muscle of WT and *Tph1* MKO mice after 12 weeks of high-fat diet

(HFD) feeding. Log2 fold change values are plotted on the x-axis and gene names on the y-axis. Positive values indicate upregulation and negative values indicate downregulation. Cutoff, $P < 0.05$ and $|\text{log2 Fold change}| > 1.0$. (b) Gene set enrichment analysis for selected biological processes. The top enriched terms include "long-chain fatty acid transport", "fatty acid transport", "glycerophospholipid biosynthetic process", and "receptor signaling pathway via JAK-STAT". (c) Functional categorization of DEGs by GO terms. Bar graphs show the distribution of DEGs in three categories: Biological Process (BP, red), Cellular Component (CC, blue), and Molecular Function (MF, green).

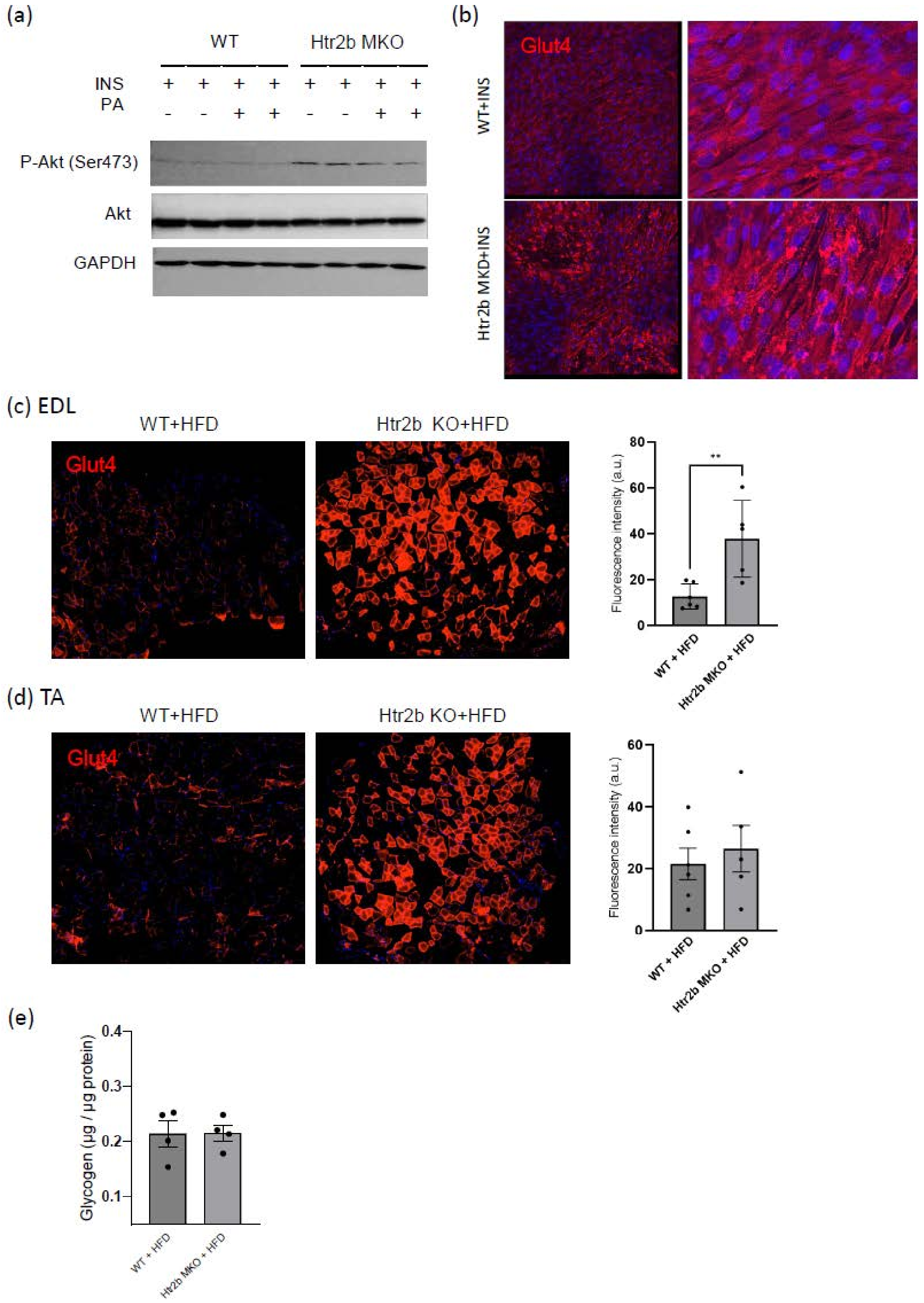

Supplementary Fig. 3. (a) Western blot images showing phosphorylated Akt (P-Akt, Ser473), total Akt and GAPDH levels of C2C12 myotubes were examined after 15 minutes treatment

with or without 100nM insulin and palmitic acid (PA, 0.25mM, 48 hours pre-treatment). (b) Immunofluorescence staining for Glut4 (red) from WT and *Htr2b* KO C2C12 myotubes 30 minutes after 100nM insulin treatment. Cell nuclei are stained with DAPI (blue). (c, d) Glut4 immunofluorescence in extensor digitorum longus (EDL) muscle sections (c) and tibialis anterior (TA) muscle sections (d) from WT+HFD (high fat diet) and *Htr2b* MKO+HFD mice. (e) Glycogen levels of quadriceps muscle from WT+HFD and *Htr2b* KO+HFD mice. ***P*-value<0.01

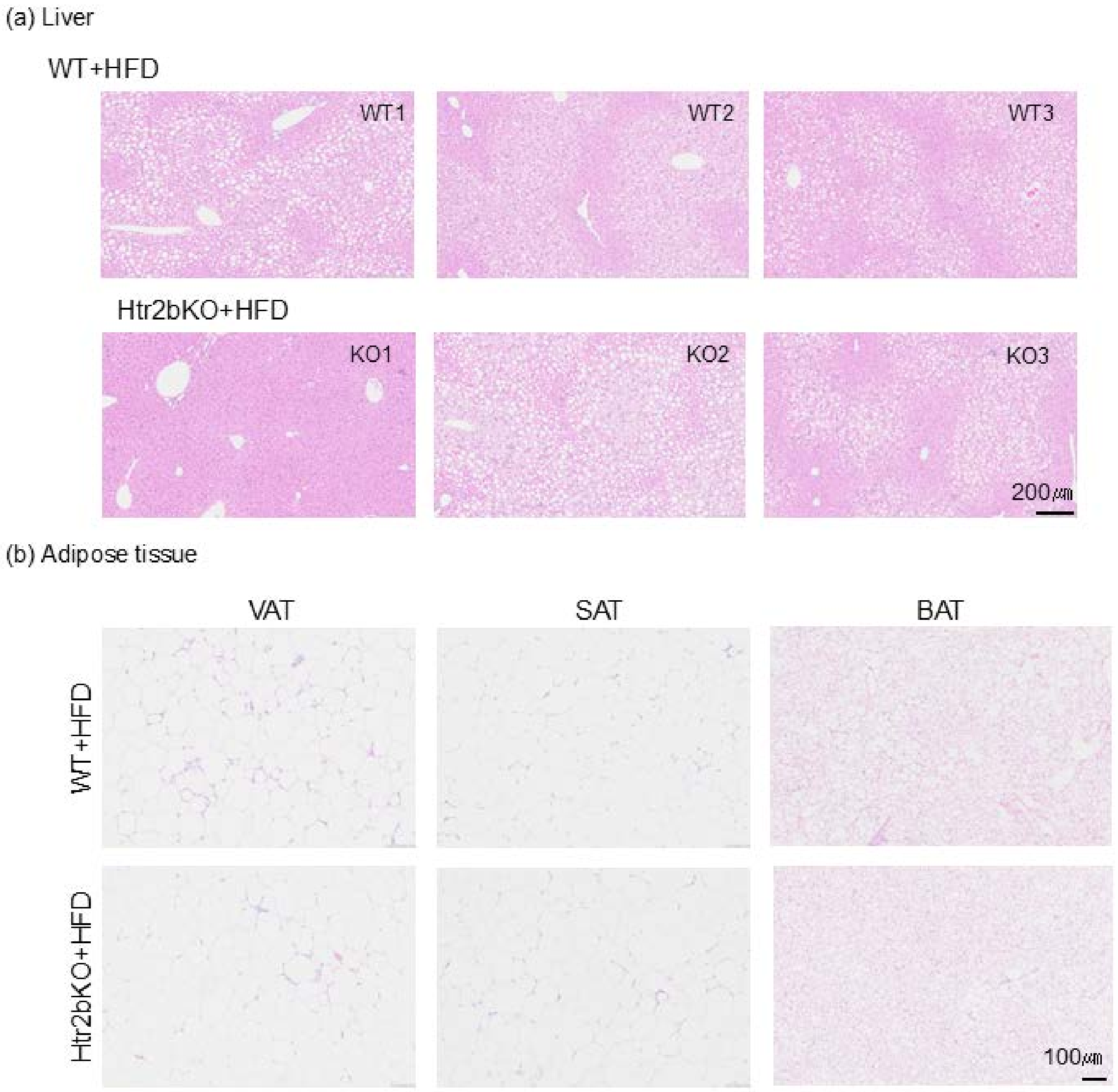

Supplementary Fig. 4. Representative H&E staining images of liver (a) and adipose tissues (b) of *Htr2b* MKO mouse after 8 weeks of high fat diet feeding. KO, knock-out; VAT, visceral adipose tissue; SAT, subcutaneous adipose tissue; BAT, brown adipose tissue.

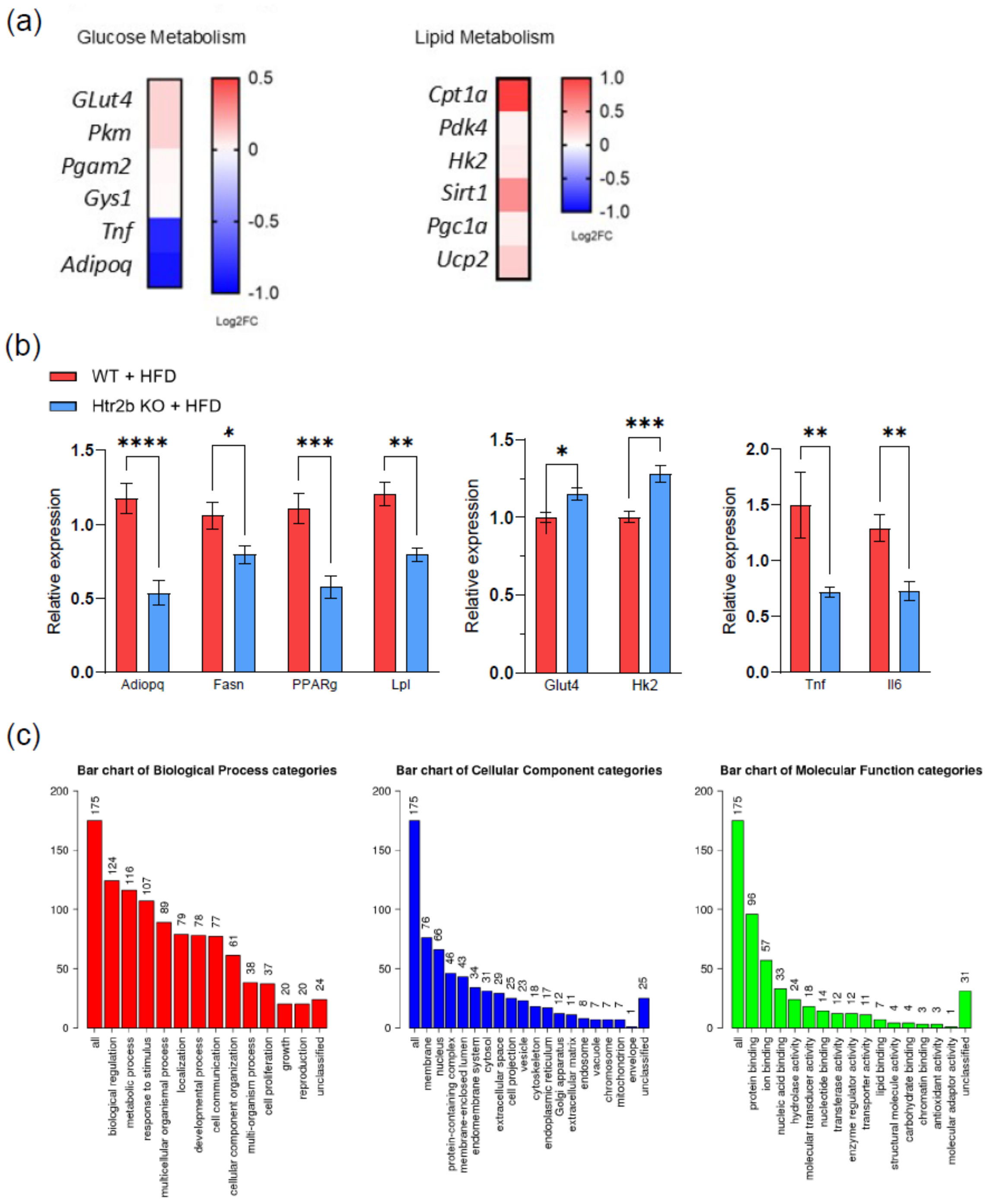

Supplementary Fig. 5. (a) Heatmaps of DEGs involved in glucose metabolism (left) and lipid metabolism (right). Genes are plotted based on log2 fold change (Log2FC), with upregulated genes in red and downregulated genes in blue. (b) The mRNA expression of the indicated genes was determined by real-time PCR in quadriceps muscles from WT and *Htr2b* MKO mice. (c) Gene ontology (GO) analysis of DEGs across biological processes (BP, red), cellular components (CC, blue), and molecular functions (MF, green).

Supplementary Table 1. List of top 10 up-regulated and top 10 down-regulated DEGs genes from DEG analysis *Htr2b* MKO mouse muscle compared to WT mouse

Gene name	Log2 FC	p-value	Adj.p-value
**Up-regulated genes**			
Cfap91	6.457092144	1.96E-08	8.41E-06
Catsperg1	2.114039279	4.21E-08	1.58E-05
Actc1	1.9098244	7.42E-114	1.08E-109
Shisal1	1.791572516	5.99E-07	0.000156045
Sln	1.523721057	6.79E-10	5.21E-07
Plekhh1	1.399361731	1.58E-05	0.002209143
Gm10851	1.289951299	9.16E-07	0.000208793
Igfn1	1.215757931	1.38E-05	0.002078647
Ighg2c	1.121340583	5.68E-12	1.04E-08
Myh8	1.078054266	1.45E-06	0.000312223
**Down-regulated genes**			
Ubd	-3.476773317	3.76E-10	3.43E-07
Tph2	-3.149156595	3.41E-08	1.34E-05
Ffar2	-1.960001938	5.59E-06	0.000982094
1700047G03Rik	-1.716289856	1.06E-05	0.001667213
Dkk3	-1.538540687	1.36E-43	9.94E-40
Cd28	-1.204868901	1.74E-08	7.92E-06
Tshr	-1.202449274	8.85E-06	0.001419235
Cdh4	-1.054225011	4.11E-07	0.000113095
Serpina3c	-0.914232149	4.27E-06	0.000798024
Aldh1a7	-0.905716563	3.56E-05	0.004361912
